# Digital Characterization of Clinical Subtypes of Oral Lichen Planus by Means of a Semi-Automated Morphometric Analysis: A Retrospective Observational Study

**DOI:** 10.3390/diagnostics15243217

**Published:** 2025-12-16

**Authors:** Keren Martí De Gea, Eduardo Pons-Fuster, Pia López-Jornet

**Affiliations:** 1 Dentistry Clinic, Department of Dermatology, Stomatology, Radiology and Physical Medicine, Faculty of Medicine, Hospital Morales Meseguer, University of Murcia, Marques Velez S/N, 30008 Murcia, Spain; k.martigea@um.es; 2 Human Anatomy Department, IMIB Biomedical Research Institute of Murcia (IMIB), University of Murcia, Campus Mare Nostrum, 30120 Murcia, Spain; eduardo.p.f@um.es

**Keywords:** oral lichen planus, histomorphology, clinical phenotypes, semi-automated learning, nuclear morphology

## Abstract

**Background**: Oral lichen planus (OLP) is a chronic inflammatory disease of unknown etiology. Its clinical and histopathological diagnosis remains challenging due to the variability of its manifestations and the subjectivity involved in interpretation. **Objective**: This study aimed to examine the relationship between different clinical phenotypes of OLP (reticular, erosive, and mixed) and histomorphological features obtained through digital analysis with semi-automated segmentation. **Methods**: A retrospective review of 100 OLP cases was conducted. Clinically, the samples were classified into three groups: 68 reticular, 16 erosive, and 16 mixed. Epithelial and connective tissue parameters were evaluated on hematoxylin–eosin-stained sections using digital tools and segmentation algorithms. **Results**: The erosive phenotype showed greater irregularity of suprabasal nuclei (*p* = 0.008) and a higher basal nucleus-to-cytoplasm ratio (*p* = 0.02). No significant differences were found among the groups regarding epithelial thickness or lymphocyte density (*p* > 0.05). **Conclusions**: The cellular alterations observed in the erosive subtype may reflect higher tissue activity and provide additional elements for its characterization. Digital morphometric analysis appears to be a promising complementary tool, although further studies are needed to confirm its diagnostic applicability.

## 1. Introduction

Oral lichen planus is a chronic inflammatory disease that affects the oral cavity, skin and mucosal surfaces, and has been classified by the World Health Organization (WHO) as a potentially malignant oral lesion (PMOL) [1]. This condition does not have a completely clear etiology [2,3].

Its worldwide prevalence is estimated to be around 1%, with a range that varies between 0.1% and 5% according to the region, being more frequent in Europe and less common in Asia and America. It mainly affects middle-aged women, with a rise in appearance between the fourth and sixth decades of life [3,4]. Its relative frequency, combined with its potential for malignant transformation [1,2,3], fully justifies the need to continue its in-depth study.

Distinguishing OLP from other similar lesions is important for estimating the risk of malignant transformation, as well as to guide its treatment [2,5,6,7]. However, its diagnosis is not simple, as it depends on both clinical and histopathological criteria, and one of the greatest challenges comes from the difficulty in harmonizing both approaches [1,2,3].

From a clinical point of view, the aim is to classify lesions into subtypes, such as reticular, plaque, atrophic, erosive–ulcerative and bullous. To simplify the diagnosis and to reduce the interpretative complexity, these subtypes are commonly grouped into three main categories: reticular lesions, erosive lesions, and mixed lesions. This categorization is clinically relevant, as the different forms vary in symptom severity and therapeutic needs, and, in some cases, have been associated with distinct risk profiles [5,6,8].

On the other hand, from the histopathological perspective, the approach is directed towards confirming or discarding the presence of OLP. According to internationally accepted histopathological features, as outlined in the consensus report from the WHO Collaborating Centre for Oral Cancer, oral lichen planus is characterized by a band-like lymphocytic infiltrate, hydropic degeneration of basal cells, lymphocytic exocytosis, and absence of the epithelium [1]. However, despite the criteria being well-defined, many studies have shown a considerable inter-observer and intra-observer variability in the interpretation of histological samples [5,7,9,10,11]. Beyond distinguishing OLP from other conditions, it would also be valuable to determine whether microscopic examination can consistently reveal differences between its clinical subtypes (reticular, erosive and mixed), as these forms may reflect distinct underlying tissue responses. Identifying reproducible microscopic patterns would provide clinicians with objective cues to support subtype classification. In this context, quantitative digital morphometric analysis offers a means to define measurable morphological parameters and to detect subtle structural variations that may escape routine microscopy, thereby reducing subjectivity and contributing to a more standardized evaluation of OLP.

We hypothesized that the different clinical phenotypes of OLP present subtle structural differences that can be detected and quantified through digital morphometric analysis. The objective of the study was assess if there are morphometric differences between the main clinical phenotypes of oral lichen planus, through the quantitative analysis of digitalized histological images and a trained classifier to segment and classify tissue structures.

## 2. Materials and Methods

### 2.1. Study Design

The present retrospective consecutive observational study was conducted with a sample of 100 patients who had been previously diagnosed with oral lichen planus (OLP). The cases were identified through a review of the department’s histopathology archive, which was carried out between January 2024 and January 2025. The cases were selected from the archives of the Department of Oral Medicine of the Faculty of Medicine at the University of Murcia and reported in accordance with the STROBE (Strengthening the Reporting of Observational Studies in Epidemiology) guidelines for observational studies [12]. 

The diagnosis of OLP was established following the clinicopathological criteria proposed by Van Der Meij and Van Der Waal (2003) [10]. The study was approved by the Ethics Committee of the University of Murcia (reference: M10/2023/108; Date of approval: 21 February 2025) and all participants signed the corresponding informed consent, in accordance with the ethical principles of the Declaration of Helsinki [13].

#### 2.1.1. Case Selection

Cases were selected from the department’s histopathology archive according to the following criteria:

1. Inclusion criteria:
  1.1 Confirmed clinical and histological OLP diagnosis following Van Der Meji and Van Der Waal (2003) [10]. Clinical evaluations were performed by an experienced oral medicine specialist and histopathological assessments by an experienced oral pathologist;
  1.2 Hematoxylin–eosin-stained biopsies available in digital format;
  1.3 Complete clinical and histopathological information recorded in the medical record.
2. Exclusion criteria:
  2.1 Patients who were minors;
  2.2 Patients under immunosuppressive or steroid treatment at the time of biopsy;
  2.3 Samples with evidence of epithelial dysplasia;
  2.4 Cases with histological or clinical features indicative of oral lichenoid lesions (OLLs), including drug reactions or contact reactions.

Together, these inclusion and exclusion criteria ensured that all selected cases met well-established diagnostic standards and provided a consistent and reliable sample for analysis.

**Figure 1:**
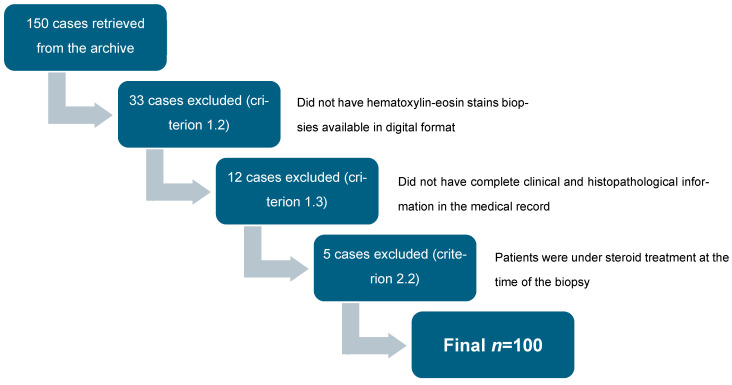
Flow diagram of case selection, adapted from the STROBE statement [12].

From an initial pool of 150 cases retrieved from the archive, 33 were excluded for not meeting inclusion criterion 1.2, 12 for not meeting criterion 1.3, and 5 for not meeting criterion 2.2, resulting in 100 eligible cases for analysis (Figure 1).

There were no missing data for any of the variables, as only patients meeting all inclusion criteria and with complete clinical and histopathological records were selected.

#### 2.1.2. Sample Size

A previous sample calculation was not performed, as the study was conducted on an existing database. A non-probabilistic convenience sampling method was used. All eligible cases available in the archive during the study period and meeting the predefined inclusion criteria were included, with no additional clinical or subjective considerations influencing case selection. This approach ensured that the sample reflected the cases routinely biopsied and recorded in the department. The final sample was composed of 100 cases, distributed into three groups according to the clinical phenotype: reticular (*n* = 68), erosive (*n* = 16), and mixed (*n* = 16).

### 2.2. Gathering of Clinical Data

The clinical variables used were collected through a review of the previously recorded medical histories and are summarized in Table 1. The following were included in this group: the OLP phenotype (reticular, erosive, and mixed), the anatomical localization from which the diagnostic biopsy was taken (buccal mucosa, palate, floor of the mouth, labial mucosa, gums and tongue), age, and sex. For the purposes of this study, reticular lesions were defined by the presence of characteristic white striae, erosive lesions by the presence of epithelial erosion or ulceration, and mixed lesions by the concurrent presence of both reticular areas and erosive or atrophic areas in the same patient at the time of diagnosis.

### 2.3. Gathering of Histologial Data

Histological variables were determined from the analysis of digitalized hematoxylin–eosin-stained sections, through the use of the QuPath (version 0.5.1) and Fiji (ImageJ 1.54p) software programs, and are also detailed in Table 1.

The digitalization of the slides was performed by using a Leica DM6200 microscope coupled to a Leica DFC280 camera (Leica Microsystems, Wetzlar, Germany), which was connected to a computer, with the acquisition software Leica Application Suite X, version 3.7.5.24914 (Leica Microsystems CMS GmbH, 2021). 10× objective lenses were used.

The analysis was performed following a standardized protocol to ensure the consistency in the segmentation, quantification, and measurement of the different epithelial and connective components.

#### 2.3.1. Assessment of Reproducibility

All quantitative histological measurements were performed under blinded conditions, as the observers had no access to the clinical subtype of each case. To assess measurement reliability, a quality-control analysis was performed on 40% of the sample. Intra-observer agreement was evaluated by repeating the measurements after a two-week interval, and inter-observer agreement was assessed by a second independent observer. Agreement for both analyses was quantified using the intraclass correlation coefficient (ICC), which reached 0.75, a value considered indicative of good reproducibility in morphometric studies.

#### 2.3.2. Epithelial Stratum

**Figure 2:**
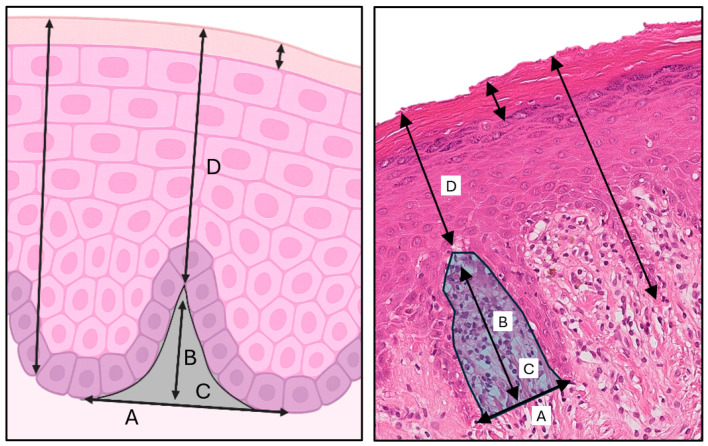
Schematic illustration (**left**) and corresponding histological section (**right**) showing the epithelial and interpapillary valley measurements analyzed in this study. Parameters include: (A) width, (B) depth, (C) area, and (D) height.

In each biopsy, five randomly selected epithelial thicknesses, defined as the perpendicular distance from the basal membrane to the epithelial surface, were measured (Figure 2). In parallel, five measurements of the thickness of the keratin layer (Figure 2) were recorded. The interpapillary valleys, defined as the connective tissue spaces delimited by the adjacent epithelial processes, were also analyzed. In each sample, five representative valleys were analyzed, in which the following parameters were studied (Figure 2):

- Width: horizontal distance (μm) between the apices of two adjacent epithelial processes, drawn as an imaginary baseline.
- Depth: perpendicular distance (μm) from this baseline to the deepest point of the connective tissue within the valley.
- Area: total surface area (in μm^2^) manually delimited following the epithelial contours and the base of the valley.
- Height: distance (in μm) from the deepest apex of the valley to the furthest point in the perpendicular direction from the epithelial surface.

All epithelial and interpapillary measurements were performed manually in QuPath using the built-in measurement tools. No automated or machine-learning segmentation classifier was used for these parameters.

#### 2.3.3. Connective Stratum

In the underlying stroma, the lymphocytes and blood vessels were quantified. In the case of the blood vessels, the structures located within the five circles with an area of 45,000 µm^2^, which were randomly distributed around the entire connective tissue, were quantified.

**Figure 3:**
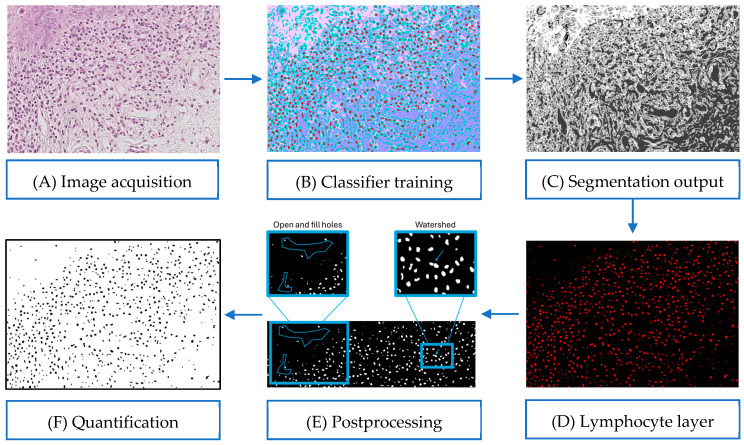
Semi-automated workflow for lymphocyte quantification. (**A**) Original H&E-stained image. (**B**) Manual annotation of histological classes in LABKIT. (**C**) Initial segmentation output. (**D**) Binary mask isolating lymphocytes. (**E**) Postprocessing to refine segmentation. (**F**) Final mask used for automated quantification.

In turn, the quantification of the lymphocytes was performed through a semi-automated analysis pipeline structured into six consecutive stages, which included the acquisition of images, until the final quantification, as summarized in Figure 3.

The process began with obtaining digital images from histological sections stained with hematoxylin–eosin. For each patient, three micrographs were selected, which were representative of the sub-epithelial area (50 µm scale, approximate area of 150.000 µm^2^ per image). These images were extracted as regions of interest (ROI) of the complete digital biopsies and exported in TIFF format with a physical resolution of 0.25 µm/pixel, equivalent to an area of 0.0625 µm^2^ per pixel (Figure 3A).

Next, the classifier was trained using the LABKIT plugin from Fiji/ImageJ, based on automated learning supervised by the random forest algorithm [14]. A total of 30 representative images were selected for the training phase, within which five histological classes were defined through manual annotations (scribbles), with the lymphocytes being the class of interest (Figure 3B).

After completing the training, the system generated preliminary segmentations of the histological structures, allowing for the visualization of the lymphocytes as independent objects within the tissue. These segmentations were iteratively adjusted until reaching a morphologically coherent classification (Figure 3C).

Once the classifier was validated, its application was automated for the complete set of images (*n* = 300) through a personalized macro developed with the IJM language. The “lymphocytes” layer was isolated as a specific binary layer which served as the basis for the posterior quantitative analysis (Figure 3D).

Posteriorly, a post-processing pipeline was then applied to the resulting binary masks. This included the application of the commands Open, Fill Holes, and Watershed, to eliminate fine noise, close incomplete contours, and separate adjacent lymphocytes, respectively (Figure 3E).

Lastly, the automated quantification was performed through the Analyze Particles module, establishing a minimum area threshold of 25 µm^2^ and excluding the objects in contact with the image borders. The total number of individual lymphocytes per image was exported in .csv format, and the lymphocyte density per patient was calculated as the mean of the three counts obtained (Figure 3F).

#### 2.3.4. Cellular Histomorphology

**Figure 4:**
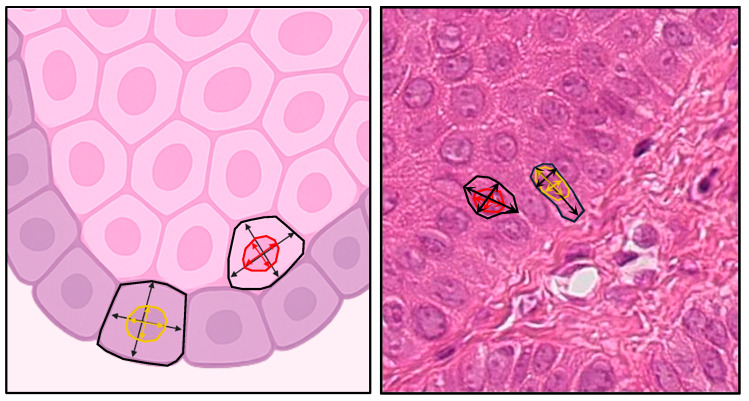
Illustration (**left**) and histological section (**right**) showing cellular and nuclear morphometric parameters measured in basal and suprabasal epithelial cells.

To analyze the cellular morphology, the perimeters and areas of five basal cells and five suprabasal cells were measured (Figure 4). The roundness formula was taken into account to analyze and interpret the results obtained.

$$Roundness\frac{{\left(perimeter\right)}^{2}\times 1000}{\left(4\times \pi \times area\right)}$$

- 1 = perfect circle;
- >1 = Oblong and non-circular objects.

In addition, the method of direct proportion of areas was used to relate the area of the cytoplasm and the nucleus, in both suprabasal and basal cells.

$$\%\;Nucleus=\frac{area\;of\;the\;nucleus}{area\;of\;the\;cytoplasm\times 100}$$

All cellular and nuclear measurements were performed manually in QuPath using the built-in measurement tools, without the use of automated segmentation or classifier-based approaches.

### 2.4. Statistical Analysis

The statistical analysis was performed with R software (version 4.4.2). Data normality was assessed using the Shapiro–Wilk test. For continuous variables, differences between OLP clinical groups were evaluated using one-way ANOVA; when the overall ANOVA was significant, pairwise group comparisons were performed with Tukey’s post hoc test to adjust for multiple comparisons within each variable. For categorical variables the assumptions of the Chi-square test were not met due to the presence of sparse cells with expected frequencies below 5. Therefore, associations between clinical phenotype and age group, sex, and anatomical location were re-analyzed using Fisher’s exact test, applying Monte Carlo simulation for contingency tables larger than 2 × 2. *p*-values were adjusted for multiple comparisons across variables using Holm’s method. A *p*-value < 0.05 was considered statistically significant after the corresponding adjustment.

## 3. Results

The data supporting the findings of this study are available within the article. Further details are available from the corresponding author upon request.

### 3.1. Clinical Data

One hundred patients with OLP were included: 68 reticular, 16 erosive, and 16 mixed. Statistically significant associations were found between the clinical type and age, and sex and the anatomical location, as shown in Table 2.

### 3.2. Histological Data

#### 3.2.1. Epithelia Stratum

The analysis performed between the epithelial parameters and different clinical phenotypes did not show statistically significant differences (Table 3).

#### 3.2.2. Connective Stratum

Statistically significant differences were identified in the percentage of blood vessels between the different clinical phenotypes. In turn, no statistically significant differences were observed in the number of lymphocytes between groups (Table 4).

#### 3.2.3. Cellular Histomorphology

Statistically significant differences were observed in two nuclear parameters between the clinical phenotypes, specifically in the suprabasal nuclei and in the nucleus–cytoplasm ratio in the basal cells. The remaining variables analyzed did not show relevant differences between groups (Table 5).

## 4. Discussion

Oral lichen planus (OLP) is a potentially malignant lesion with different clinical variants in their presentation and possibly in their biological behavior [2,3,4,7]. In the present study, quantifiable structural differences were identified between the main clinical phenotypes of OLP, particularly in the erosive type, which showed a higher nuclear roundness in the suprabasal cells and a higher nucleus–cytoplasm ratio in the basal layer. These findings could indicate a more active tissue response, associated with persistent chronic inflammation, and could alert to increased tissue activity.

From the clinical perspective, associations were confirmed between the OLP subtypes and variables such as age and anatomical location. The pattern observed (reticular predominated in patients aged from 51 to 60 years old, the erosive type in patients predominantly between 41 and 60 years, and the mixed in those older than 70) coincides with that reported by Sanches et al. [6], as well as with the female predominance and the anatomical distribution described in other studies [5,7,8]. These data support the idea that the clinical phenotypes represent not only morphological variations, but different manners of expression of the same inflammatory process.

As for the conventional histological analysis, no significant differences were found in variables such as epithelial thickness or keratinization pattern, which coincides with that described by other authors [6,8,15]. Although the overall structural changes did not show differences, the quantitative analysis showed discrete but consistent cellular alterations, such as the scarce nuclear roundness in the suprabasal layer and the increase in the nucleus–cytoplasm ratio in the erosive-type basal layer. These changes, which are difficult to perceive through visual observation, reinforce the value of digital measurements as objective complements as compared to the subjectivity of manual analysis [16].

The higher nucleus–cytoplasm ratio may be due to phenomena such as hydropic degeneration, frequent in OLP, which reduces cytoplasmic volume due to vacuolization [3,5,8,17]. This mechanism has been described by Mutafchieva [9] as part of the cellular damage associated with persistent inflammation. It could also indicate cell turnover, although in this series, no increased mitosis was observed, and neither signs of dysplasia. As Ghazi et al. [18] warn, these changes must be interpreted in an inflammatory context, not as markers of malignant transformation.

Although no statistically significant differences were found in the density of the inflammatory infiltrate, a trend was observed towards a higher number of lymphocytes in the erosive group, similar to those described by Akbari et al. [19], who reported a more marked immunological response.

As for vascularization, the finding of a lower proportion of vessels in the erosive group seems contradictory given its higher inflammatory activity. However, this result must be interpreted as a technical limitation of the hematoxylin–eosin staining, which does allow for the precise identification of functional microvasculature. Studies such as those by Shrikaar et al. [20], which use immunohistochemical markers such as CD34, have shown a higher vascularization in erosive lesions.

In this sense, the results are in agreement with Idrees et al. [21] in that the morphometric analysis can provide valuable information, which has been demonstrated by recent studies that apply artificial intelligence to analyze histological characteristics [22,23,24]. In line with this evidence, the alterations observed in the erosive pattern could become a useful complement for the clinician, both to facilitate therapeutic and follow-up decisions and to support future prognostic assessment by helping identify patients who may present more active or evolving disease patterns. Nevertheless, prospective studies are required to confirm their usefulness in real-world clinical contexts. Furthermore, the standardized nature of these measurements makes them suitable for future AI-assisted tools aimed at enhancing diagnostic consistency and streamlining the evaluation process.

It is important to clarify that although automated segmentation was used to quantify cellular structures, this tool was not used with diagnostic or classificatory aims. Thus, no metrics were calculated, such as sensitivity or ROC curves. However, its application allowed for reducing the variability of manual analysis and standardizing the measurements, especially in subtle morphological parameters.

Among the strengths of the study, the following stand out: the rigorous selection of the cases, the structured quantitative approach, the use of reproducible tools that were exclusively applied to OLP and the examination of quantitative differences among OLP subtypes, providing a complementary angle to the literature, which has primarily addressed distinctions between OLP and lichenoid lesions [5,6,16,17,18]. To our knowledge, few studies have explored subtype-specific morphometric patterns within OLP itself, which constitutes a distinctive contribution of the present work. As limitations, we recognize the small sample size in some of the subgroups, the exclusive use of HE stain for vascular evaluation, and the lack of a longitudinal clinical correlation. The unequal distribution of clinical subtypes in our sample reflects the real epidemiological pattern of OLP, in which reticular forms are substantially more common than erosive or mixed presentations. However, this natural imbalance may have reduced the statistical power to detect subtle differences in the smaller subgroups, meaning that some findings might not have reached significance due to limited sample size rather than the absence of a true effect. Additionally, potential confounding by age or sex cannot be fully excluded, as no adjusted analyses were performed; therefore, minor unmeasured differences in these variables may have had a slight influence on some of the morphometric outcomes. Finally, the retrospective single-center design and the fact that all cases originate from a single geographical region may limit the generalizability of the results to wider populations.

## 5. Conclusions

The present study demonstrates that the morphometric analysis can detect specific cellular alterations between clinical subtypes of OLP, especially the erosive type, even when the general epithelial architecture does not show changes. This reinforces its usefulness as a complementary tool in contexts of uncertain diagnosis. Although no overall structural differences were found, the digital morphometry allowed detection of specific cellular changes in the erosive subtype, which reinforces its potential as a complementary diagnostic tool.

These findings support the added value of digital morphometry as a complementary approach, offering greater objectivity and sensitivity to subtle structural variations that may not be consistently detected through conventional histopathological assessment.

## Figures and Tables

**Table 1:** Clinical and histological variables included in the study.

Variable	Definition	Measurement/Source	Units/Categories	Notes
**OLP clinical phenotype**	Clinical presentation of the lesion at diagnosis, grouped into main categories	Recorded in clinical chart; classified by oral medicine specialist as reticular, erosive, or mixed	Reticular/Erosive/Mixed	—
**Anatomical localization**	Intraoral site from which the diagnostic biopsy was taken	Medical record and histopathology request form	Buccal mucosa/Palate/Floor of mouth/Labial mucosa/Gingiva/Tongue	Only the biopsy site was coded for this variable
**Age**	Patient’s age at time of biopsy	Medical record	<30/30–40/41–50/51–60/61–70/>70	Also grouped into age ranges for descriptive analysis
**Sex**	Biological sex recorded in medical record	Medical record	Female/Male	—
**Epithelial thickness**	Perpendicular distance from basal membrane to epithelial surface	Measured in QuPath on 5 randomly selected fields per case	Micrometers (µm)	Mean of 5 measurements per case
**Keratin layer thickness**	Thickness of superficial keratinized layer	Measured in QuPath on 5 randomly selected fields per case	Micrometers (µm)	Mean of 5 measurements per case
**Interpapillary valley parameters**	Morphology of connective tissue spaces between epithelial ridges	Measured in QuPath: width, depth, area, height	Micrometers (µm) for width, depth, height; square micrometers (µm^2^) for area	Mean of 5 valleys per case
**Blood vessel proportion**	Percentage of stromal area occupied by blood vessels	Manual count in 5 circles of 45,000 µm^2^ per case	%	Identified on H&E-stained sections
**Lymphocyte count**	Density of lymphocytes in subepithelial stroma	Semi-automated segmentation and particle count in Fiji/ImageJ	Number of lymphocytes per image	Mean of 3 images per case
**Nuclear roundness (basal/suprabasal)**	Shape descriptor: 1 = perfect circle; >1 = elongated	Perimeter and area measured in Fiji/ImageJ; roundness formula applied	Dimensionless ratio	Mean of 5 cells per layer per case
**Nucleus–cytoplasm ratio (basal/suprabasal)**	Relative area of nucleus to cytoplasm	Direct proportion of measured areas	%	Mean of 5 cells per layer per case

**Table 2:** Clinical data and OLP phenotypes. Data are presented as number of cases. *p*-values from Fisher’s exact test (Monte Carlo simulation for tables >2 × 2); values adjusted for multiple comparisons using Holm’s method. * Statistically significant (*p* < 0.05).

		Reticular	Erosive	Mixed	*p*-Value
Age	<30	2	0	0	0.042 *
	30–40	6	0	2	
	41–50	8	6	2	
	51–60	32	4	4	
	61–70	14	4	2	
	>70	6	2	6	
Sex	Female	48	14	16	0.024 *
	Male	20	2	0	
Anatomical localization	Labial mucosa	2	0	0	0.006 *
	Buccal mucosa	52	6	12	
	Palate	2	0	0	
	Gum	6	10	4	
	Tongue	4	0	0	
	Floor of mouth	2	0	0	

**Table 3:** Epithelial stratum and OLP phenotypes. Data are presented as mean values with 95% confidence intervals (µm or %). *p*-values from one-way ANOVA (no post hoc adjustment applied, as no overall significance was found).

		Reticular	Erosive	Mixed	*p*-Value
Total epithelial width (μm)		190.18 (173.72–206.64)	178.83 (155.05–202.61)	188.43 (133.09–243.77)	0.78
Keratin width (μm)		60.51 (29.68–91.34)	29.61 (17.67–41.55)	57.13 (22.46–91.80)	0.39
% Epithelium affected by crests		8.96 (5.91–12.01)	8.78 (5.42–12.14)	7.49 (2.45–12.53)	0.83
Valleys	Depth (μm)	122.73 (105.95–139.51)	118.62 (80.45–156.79)	128.66 (81.51–175.81)	0.93
	Width (μm)	145.89 (125.71–166.07)	118.58 (79.08–158.08)	152.27 (113.75–190.79)	0.42
	Area (μm^2^)	11,283.53 (7846.07–14,720.99)	9341.58 (4967.48–13,715.68)	11,503.77 (5060.25–17,947.29)	0.86
	Height (μm)	230.51 (206.99–254.03)	218.83 (175.10–262.56)	268.28 (186.91–349.65)	0.36

**Table 4:** Connective stratum and OLP phenotypes. Data are presented as mean values with 95% confidence intervals (%, counts). *p*-values from one-way ANOVA; for parameters with overall significance, pairwise comparisons were adjusted using Tukey’s post hoc test. * Statistically significant (*p* < 0.05). Different superscript letters indicate statistically significant differences between groups in post hoc comparisons (Tukey’s test, *p* < 0.05); groups sharing at least one letter do not differ significantly.

	Reticular	Erosive	Mixed	*p*-Value
% Blood vessels	0.23 ^b^ (0.21–0.25)	0.18 ^a^ (0.14–0.22)	0.24 ^ab^ (0.20–0.28)	0.03 *
Number of lymphocytes	523 (443.85–602.15)	641 (445.39–836.61)	548 (319.85–776.15)	0.49

**Table 5:** Cell histomorphology and OLP phenotypes. Note: A roundness greater than 1 indicates a more elongated shape, while values closer to 1 reflect spherical nuclei. Data are presented as mean values with 95% confidence intervals (ratios or %). *p*-values from one-way ANOVA; for parameters with overall significance, pairwise comparisons were adjusted using Tukey’s post hoc test. * Statistically significant (*p* < 0.05). Different superscript letters indicate statistically significant differences between groups in post hoc comparisons (Tukey’s test, *p* < 0.05); groups sharing at least one letter do not differ significantly.

	Reticular	Erosive	Mixed	*p*-Value
Roundness of the nucleus of the suprabasal cells	1.38 ^b^ (1.36–1.40)	1.32 ^a^ (1.23–1.35)	1.39 ^b^ (1.33–1.45)	0.008 *
Roundness of basal cell nucleus	1.56 (1.53–1.59)	1.55 (1.47–1.63)	1.52 (1.47–1.57)	0.30
% Suprabasal nucleus–cytoplasm ratio	29.24 (28.14–30.34)	28.01 (25.79–30.23)	27.59 (25.82–29.36)	0.12
% Basal nucleus–cytoplasm ratio	42.70 ^ab^ (41.15–44.25)	43.99 ^b^ (41.64–46.35)	39.81 ^a^ (36.50–41.18)	0.02 *

## Data Availability

The data generated in this study are available upon request from corresponding author. To protect privacy, the data are not publicly available.
